# The Mediating Role of Stress in the Relationship Between Attention to Threat Bias and Psychotic-Like Experiences Depends on Coping Strategies

**DOI:** 10.3389/fpsyt.2020.00307

**Published:** 2020-04-29

**Authors:** Katarzyna Prochwicz, Joanna Kłosowska, Aleksandra Dembińska

**Affiliations:** ^1^Institute of Psychology, Jagiellonian University, Krakow, Poland; ^2^Department of Psychology, Pedagogical University, Krakow, Poland

**Keywords:** psychotic-like experiences, cognitive biases, stress, coping, mediation, moderation

## Abstract

**Aim:**

Recent studies have provided evidence that enhanced stress level is associated with the increase of psychotic symptoms in both clinical and non-clinical populations. It has also been demonstrated that cognitive biases contribute to psychotic experiences. However, it remains unclear whether the effect of cognitive biases and perceived stress on psychotic-like experiences (PLEs) is influenced by coping methods. In the present study we examined whether the relationship linking cognitive biases with PLEs is mediated by the level of stress and whether particular coping methods modify the relationship between stress and PLEs.

**Methods:**

The study sample consisted of 290 non-clinical subjects; study variables were assessed by questionnaires. Moderated mediation analyses were conducted.

**Results:**

Perceived stress was found to serve as a partial mediator in the relationship linking attention to threat (ATB) and external attribution biases (ETB) with psychotic-like experiences. Also, moderated mediation analysis revealed that the indirect effect of attention to threat bias on positive and depressive symptoms of psychotic-like experiences *via* perceived stress was stronger at higher levels of distraction seeking coping. Moreover, the indirect effect of ATB on depressive symptoms was moderated by task-oriented coping and emotion-oriented coping. Task-oriented coping also moderated the indirect effect of ETB on depression.

**Conclusion:**

The findings imply that both perceived stress and coping styles are important factors affecting the association between cognitive biases and psychotic-like experiences.

## Introduction

Cognitive biases have been recognized as important risk factors for various psychiatric conditions, including psychotic disorders. In line with the hypothesis of extended psychotic phenotype (1), the role of cognitive biases has been examined in samples of patients suffering from schizophrenia, individuals with ultra-high risk for psychosis (UHR), and community samples with subclinical psychotic experiences. These studies differentiated specific cognitive biases, i.e. attributional bias (2–4), attention to threat bias (5, 6), threat anticipation (7), and jumping to conclusions (8, 9) which are involved in the psychotic symptoms development. Importantly, the role of cognitive biases was confirmed not only in clinical groups, but also outside the boundaries of clinical psychosis.

Among psychological factors, heightened stress and elevated stress sensitivity have also been indicated as shaping the risk of psychosis (10–13). The central role of stress has been widely investigated and well-replicated in samples of patients with schizophrenia (13, 14), first episode psychosis (15), UHR individuals (10, 16, 17), and healthy subjects with psychosis-proneness (11, 18).

Particularly interesting, although there are only few, are studies concerning the relationship between stress and subclinical psychotic symptoms conducted on non-clinical groups. These studies to a greater extent, than research on patients with overt psychosis, give the opportunity to distinguish between the role of stress resulting from adverse life events and the role of stress being a consequence of psychotic symptoms (i.e., persecutory delusions or hallucinations). Thus, studies on non-clinical populations help to examine whether stressful life events contribute to psychosis development before its onset. Unfortunately, such studies are not only rare, but they also are focused primarily on positive symptoms (18–21). It is plausible, however, that adverse life events may be involved in the negative symptoms formation, e.g. through promoting social withdrawal or activity restriction.

A few studies have also considered the potential contribution of coping styles to the link between stress and psychosis. The majority of the research applied the distinction between adaptive and maladaptive coping. In general, task-oriented coping (focused on problem solving or cognitive reconceptualization) is viewed as adaptive, whereas emotion-oriented (focusing on emotional responses, e.g. worry, self-blame, self-preoccupation, or fantasizing) and avoidance-oriented (focusing on distraction-based activities or social diversion) coping methods are considered as less effective (19). A growing body of studies demonstrated that individuals reporting psychotic symptoms tend to use maladaptive coping methods to a greater extent than subjects denying psychotic experiences. Specifically, patients with schizophrenia are more likely to choose emotion-oriented strategies and less likely to engage in active problem solving when faced with stressful situations (16, 22). A similar coping pattern was observed in individuals at risk of psychosis (16, 17), and in adolescents and young adults with subclinical psychotic symptoms (18, 20). What is more, different types of non-adaptive coping were found to be associated with poor outcome in chronic schizophrenia patients (22) and UHR individuals (23), as well as with persistence of subclinical symptoms in a general population sample (18). In non-clinical adolescent a dose-response relationship was observed between emotionally driven coping and the development of subclinical symptoms (18). Contrarily, more adaptive, task-oriented coping was found to be associated with the decrease of attenuated psychotic experiences over a three-year period (18).

Among psychotic individuals stress experience is likely to be amplified by biased cognitive processes, such as oversensitivity to threat, threat anticipation, and tendency to perceive others as threatening. The relationship between cognitive biases and heightened stress has already been postulated in cognitive models of psychosis (24, 25). These models emphasize bi-directionality of this association; not only the presence of cognitive biases precedes stress, but the increased stress amplifies the tendency to search environment for threat. What is more, psychotic symptoms developed on the basis of cognitive biases and psychological distress may also be a source of further stress, threat sensitivity, and social withdrawal. However, it can be expected that enhanced stress may or may not exaggerate psychotic symptoms depending on which coping strategies are applied. Specifically, the use of task-oriented coping may break the vicious cycle linking cognitive biases, stress, and psychosis.

The aim of the current study was to explore the relationship between cognitive biases (attention to threat and external attribution biases), perceived stress, coping styles, and psychotic-like experiences in a non-clinical sample. Basing on the results of previous studies three hypotheses were formulated: 1) the relationship linking attention to threat bias (ATB) and external attribution bias (ETB) with psychotic-like experiences is mediated by the perceived stress; 2) the style of coping moderates the relationship between perceived stress and psychotic-like experiences; specifically, maladaptive emotion-oriented and avoidance-oriented coping increase the effect of stress on PLEs, whereas more adaptive task-oriented coping decreases the effect of stress on PLEs; 3) coping style moderates the positive indirect effect of ATB and ETB on psychotic-like experiences *via* perceived stress; in particular, this effect is stronger at higher levels of less adaptive styles of coping (emotion-oriented and avoidance oriented) and weaker at higher levels of more adaptive styles of coping (task-oriented).

**Figure 1:**
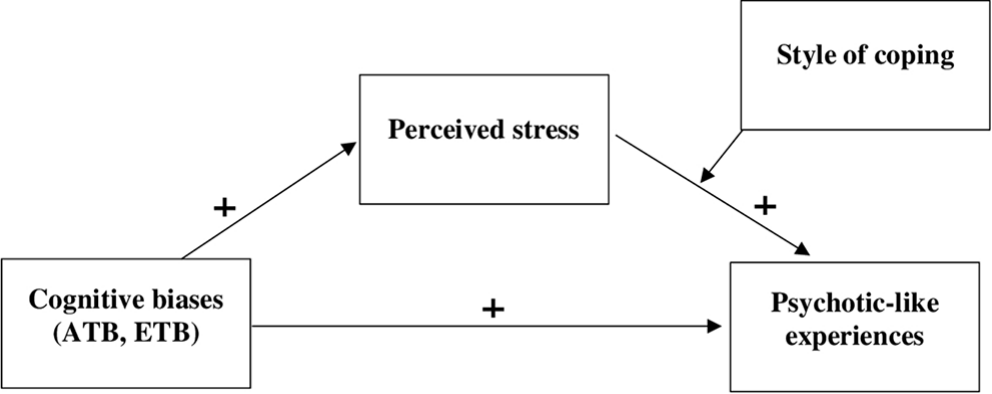
Conceptual model of moderated mediation. Note: The model assumes that attention to threat bias and external attribution bias will be positively related to perceived stress, which will be positively related to psychotic-like experiences. However, the style of coping should moderate the link between perceived stress and psychotic-like experiences, so that less adaptive styles of coping increase the effect of stress on psychotic-like experiences.

The conceptual model tested in the study is presented in **Figure 1**.

## Materials and Methods

### Participants

The initial study sample consisted of 376 participants; however, only the data obtained from participants with no history of psychiatric diagnosis (including substance abuse) and no history of clinical conditions in first and second degree relatives were included into the analyses (these data were collected with a self-report questionnaire). Therefore the final sample consisted of 290 individuals, 250 females and 39 males (one participant did not report sex) aged between 18 and 48 years (M=21.81, SD=2.72). All participants were recruited among students of the Pedagogical University of Krakow and were examined after giving the informed consent during regularly scheduled lectures. They were informed that they could refuse to participate at any time without consequences and that the study was anonymous. No form of compensation was offered as an incentive to participate. The studies were approved by the Local Ethics Committee.

### Measurements

#### Perceived Stress Scale (PSS-10)

Perceived stress was measured using the 10-item Perceived Stress Scale (PSS-10) developed by Cohen et al. (26). The Polish adaptation of the PSS-10 by Juczyński and Ogińska-Bulik (27) was utilized in the study. The scale includes six negatively phrased items that assess levels of distress and negative affect and four items that are positively phrased and reflect the perception of one's ability to deal with stressors. Each item is rated on a five-point scale from 1 (never) to 5 (very often). Participants are asked to assess the frequency of thoughts and feelings in the last month. The total score ranges from 0 to 40, with higher scores representing higher perceived stress levels (positively phrased items are reverse coded before summing the responses). Cronbach's Alpha for the scale in our sample was 0.87.

#### Coping Inventory for Stressful Situations (CISS)

Coping styles were assessed with the Coping Inventory for Stressful Situations (28). The Polish version of CISS translated and validated by Strelau et al. (29) was used in the present study. The CISS contains 48 items answered on a five-point Likert scale ranging from “never” to “very often.” The scores for three main scales measuring: task-oriented coping (16 items), emotion-oriented coping (16 items), and avoidance-oriented coping (16 items) can be calculated. Furthermore, the last scale is divided into two subscales: distraction seeking (8 items) and social diversion (5 items). Cronbach's Alpha for the current sample were: 0.90 for the emotion-oriented coping subscale, 0.88 for the task-oriented coping subscale, 0.85 for the avoidance subscale, 0.81 for the distraction seeking subscale, 0.82 for the social diversion subscale.

#### Davos Assessment of Cognitive Biases Scale (DACOBS)

DACOBS (30) is a 42-item self-report scale with seven subscales, each containing six items. Four of the subscales measure cognitive biases: jumping to conclusions (JTC), belief inflexibility (BIB), attention for threat bias (ATB), and external attribution bias (ETB). Additionally, two subscales measure cognitive limitations and one measures safety behaviors. Responses are given on a seven-point scale (1 = strongly disagree, 7 = strongly agree). Since the present study aimed at examining the role of attention to threat bias and external attribution bias in psychotic-like experiences only ATB and ETB scores were included in the analyses. The Polish translation was utilized; the Cronbach's Alpha for both ATB and ETB were 0.60 (31).

#### Community Assessment of Psychic Experiences (CAPE)

The CAPE (32) consists of 42 items assessing on a four-point Likert scale the frequency (lifetime prevalence) of psychotic-like experiences and stress induced by specific experiences. In the current study only the frequency of PLEs was considered. The CAPE distinguishes three subscales measuring different dimensions of psychotic-like experiences: positive symptoms (CAPE positive: 20 items), negative symptoms (CAPE negative: 14 items), and depression (CAPE depression: 8 items). The CAPE provides scores for each subscale, as well as a total score calculated by summarizing all scores (CAPE total). We used the Polish version of the CAPE (31); in the current study the Cronbach's alpha calculated for the total score was 0.89, for the positive symptoms subscale 0, for the negative subscale score 0, and for depression subscale 0.

## Results

### Data Analysis Plan

A correlation analysis was conducted among the variables prior to testing our hypotheses. Spearman's correlations were used to investigate the associations between the variables since some of the scores (PSS, CAPE, CISS social diversion subscale, age) deviated from normality. Point-biserial correlations were calculated to test the relationships with the “sex” variable. Proposed models were examined using the Process Macro for SPSS (33), applying models number 4 (simple mediation) and 14 (moderated mediation), with 5,000 bias corrected bootstrap samples. The variables in the models were mean centered to minimize multicollinearity. Each of the tested models examined the combination of different statistical predictors (ATB/ETB) of PLEs and different moderators (task-oriented style of coping/emotion-oriented style of coping/avoidant style of coping/distraction seeking/social diversion) of the relationship between perceived stress and PLEs. Sex and age were controlled for in the analyses. Missing data were handled with the listwise deletion (*n*=15). Bonferroni correction was not applied since it could not be assumed that the analyses were independent.

### Preliminary Analyses

**Table 1** presents the descriptive statistics of the variables and correlation matrix. Attention to threat bias and external attribution bias were positively related to psychotic-like experiences as well as to perceived stress, emotion-oriented coping style, and distraction seeking. Moreover, ETB was negatively related to coping by social diversion. Stress correlated positively with psychotic like experiences, emotion-oriented coping, and distraction seeking and negatively with task-oriented coping. Furthermore, there was a positive correlation between PLEs and emotion-oriented coping style as well as distraction seeking (with the exception of negative symptoms of PLEs which did not correlate significantly with distraction seeking). The analyses also yielded a negative, weak correlation between PLEs (with the exception of positive symptoms of PLEs) and task-oriented coping and social diversion.

**Table 1:** Descriptive statistics and results of correlational analysis (Spearman's rho).

	Min/Max	S	K	M(SD)	(2)	(3)	(4)	(5)	(6)	(7)	(8)	(9)	(10)	(11)	(12)	(13)	(14)
PSS-10 (1)	1/37	−.12	−.64	18.78(7.23)	−.17**	.64***	.11	.17**	−.11	.26**	.39***	.55***	.31***	.45***	.69***	−.03	.17**
CISS task (2)	2/80	−.13	.54	55.39(8.90)	1	−.21***	−.04	−.14*	.15*	.08	−.19**	−.12*	.05	−.19**	−.21***	.13*	.11
CISS emotion (3)	16/74	.19	−.19	43.91(11.44)		1	.25***	.30***	.00	.30***	.40***	.52***	.35***	.45***	.58***	−.09	−.17*
CISS avoidant (4)	20/72	−.24	−.31	47.32(10.12)			1	.88***	.72***	.08	.04	.06	.12*	-.05	.11	−.29***	−.19**
CISS distraction (5)	8/39	−.08	−.37	20.80(6.23)				1	.37***	.13*	.14*	.17**	.16**	.09	.20**	−.23***	−.13*
CISS social diversion (6)	6/25	−.45	−.10	17.88(4.15)					1	-.09	-.20**	-.20**	-.05	−.29***	−.18**	−.24***	−.25***
DACOBS ATB (7)	7/36	−.06	−.15	23.25(5.08)						1	.46***	.38***	.36***	.21***	.38***	−.13*	.02
DACOBS ETB (8)	7/34	.45	.30	18.58(4.67)							1	.44***	.36***	.33***	.44***	−.08	.05
CAPE total (9)	46/120	.72	.49	72.11(12.58)								1	.81***	.87***	.84***	.06	0.9
CAPE positive (10)	20/54	1.05	1.32	30.00(5.43)									1	.50***	.55***	.03	.19**
CAPE negative (11)	15/49	.65	.99	26.00(5.44)										1	.65***	.11	.12**
CAPE depression (12)	9/35	1.12	1.95	16.12(4.07)											1	−.00	−.07
Age (13)	18/48	5.63	45.67	21.18(2.72)												1	.21***
Sex (14)	–	–	–	–													1

n = 275; *p < 0.05; **p < 0.01; ***p < 0.001. PSS-10, total score of the Perceived Stress Scale; CISS task, score of subscale of the Coping Inventory for Stressful Situations measuring task-oriented coping style; CISS emotion, score of subscale of the Coping Inventory for Stressful Situations measuring emotion-oriented coping style; CISS avoidant, score of subscale of the Coping Inventory for Stressful Situations measuring avoidance-oriented coping style; CISS distraction, score of subscale of the Coping Inventory for Stressful Situations measuring distraction seeking coping style; CISS social diversion, score of subscale of the Coping Inventory for Stressful Situations measuring social diversion coping style; DACOBS ATB, Attention to Threat Bias subscale score of the DACOBS; DACOBS ETB, External Attribution Bias subscale score of DACOBS; CAPE, total score of the Community Assessment of Psychic Experiences (CAPE); women were coded as −1, men were coded as 1.

### Test of Mediation Model

The results of the analyses examining mediating effects of perceived stress in the relationship between attention to threat bias and psychotic-like experiences as well as in the linkage between external attribution bias and PLEs are presented in **Table 2**. The total effect of ATB on psychotic-like experiences (CAPE total) was positive and significant (B=0.90 SE=0.14, CI_low_=0.63; CI_high_=1.18, p<0.001) similarly as the total effect of ETB on PLEs (B=1.18, SE=0.15, CI_low_=0.89, CI_high_=1.46, p < 0.001). Furthermore, both ATB and ETB were positively related to stress, which in turn correlated (also positively) with psychotic-like experiences. After controlling for perceived stress the relationships between ATB and PLEs (B=0.54, SE=0.14, CI_low_=0.30, CI_high_=0.79, p<0.001) and between ETB and PLEs (B=0.64, SE=0.14, CI_low_=0.37, CI_high_=0.92, p<0.001) were still positive and significant, however became weaker. As expected, the indirect effect of ATB on PLEs *via* perceived stress (B=0.36, SE=0.08, CI_low_=0.22, CI_high_=0.52) as well as the indirect effect of ETB on PLEs *via* stress (B=0.53, SE=0.08, CI_low_=0.39, CI_high_=0.71) were significant suggesting partial mediations.

**Table 2:** Parameter estimates for the mediation model (dependent variable: CAPE total).

	Estimate	*SE*	Bootstrap (*n*=5,000)		
			95%CI_low_	95%CI_high_	*p*
Predictor: DACOBS ATB					
Total effect					
**DACOBS ATB→CAPE**	**0.90**	**0.14**	**0.63**	**1.18**	**<0.001**
Direct effects					
**DACOBS ATB → PSS-10**	**0.40**	**0.08**	**0.24**	**0.57**	**<0.001**
**PSS-10 → CAPE**	**0.90**	**0.09**	**0.73**	**1.07**	**<0.001**
**DACOBS ATB → CAPE**	**0.54**	**0.14**	**0.30**	**0.79**	**<0.001**
**Sex → PSS-10**	**−2.07**	**0.68**	**−3.41**	**−0.73**	**0.01**
Age → PSS-10	0.00	0.15	−0.30	0.30	0.99
**Sex → CAPE**	**4.17**	**0.98**	**2.24**	**6.11**	**<0.001**
Age → CAPE	−0.02	0.22	−0.45	0.41	0.93
Predictor: DACOBS ETB					
Total effect					
**DACOBS ETB-> CAPE**	**1.18**	**0.15**	**0.89**	**1.46**	**<0.001**
Direct effects					
**DACOBS ETB → PSS-10**	**0.65**	**0.09**	**0.48**	**0.82**	**<0.001**
**PSS-10 → CAPE**	**0.82**	**0.09**	**0.64**	**1.00**	**<0.001**
**DACOBS ETB → CAPE**	**0.64**	**0.14**	**0.37**	**0.92**	**<0.001**
**Sex → PSS-10**	**2.26**	**0.65**	**−3.53**	**−0.99**	**<0.001**
Age → PSS-10	0.03	0.14	−0.26	0.31	0.86
**Sex → CAPE**	**3.88**	**0.99**	**1.94**	**5.83**	**<0.001**
Age → CAPE	−0.03	0.22	−0.46	0.39	0.88

n = 275; R^2^ when DACOBS ATB is the predictor = 0.27, p < 0.001; R^2^ when DACOBS ETB is the predictor = 0.30, p < 0.001; PSS-10, total score of Perceived Stress Scale; DACOBS ATB, Attention to Threat Bias subscale score of the DACOBS; DACOBS ETB, External Attribution Bias subscale score for DACOBS; CAPE total, total score of the Community Assessment of Psychic Experiences; women were coded as −1, men were coded as 1.

The statistically significant results were written in bold.

A similar pattern of findings emerged when subscores of the CAPE scale were taken into account in the analyses: in each case perceived stress partially mediated the relation between cognitive bias and psychotic symptoms (see **Table 3**).

**Table 3:** Indirect effects of cognitive biases on different dimensions of CAPE.

	Estimate	*SE*	Bootstrap (*n*=5,000)	
			95%CI_low_	95%CI_high_
DACOBS ATB→CAPE total	0.36	0.08	0.22	0.52
DACOBS ATB→CAPE positive	0.08	0.02	0.04	0.14
DACOBS ATB→CAPE negative	0.14	0.03	0.09	0.21
DACOBS ATB→CAPE depression	0.14	0.03	0.08	0.19
DACOBS ETB→CAPE total	0.53	0.08	0.39	0.71
DACOBS ETB→CAPE positive	0.12	0.03	0.06	0.19
DACOBS ETB→CAPE negative	0.20	0.04	0.14	0.29
DACOBS ETB→CAPE depression	0.21	0.03	0.16	0.27

n = 275; all indirect effects are statistically significant; DACOBS ATB, Attention to Threat Bias subscale score of the DACOBS; DACOBS ETB, External Attribution Bias subscale score of the DACOBS; CAPE total, total score of the Community Assessment of Psychic Experiences; CAPE negative, negative symptoms subscale of the Community Assessment of Psychic Experiences; CAPE positive, positive symptoms subscale of the Community Assessment of Psychic Experiences; CAPE depression, depression subscale of the Community Assessment of Psychic Experiences.

### Test of Moderated Mediation Models

#### The Relationship Between Attention to Threat Bias, Stress, Coping, and PLEs

The interaction effect of perceived stress and coping on PLEs (CAPE total was significant (B=0.03, SE=0.01, CI_low_=0.001, CI_high_=0.05, p<0.05), however only in case of distraction seeking coping (**Table 4**).

**Table 4:** Parameter estimates for the moderated mediation model (dependent variable: CAPE total).

		Estimate		*SE*	Bootstrap (*n*=5,000)		
					95%CI_low_	95%CI_high_	*p*
	Mediator Variable Model – outcome: PSS-10						
**Constant**		**−10.83**		**4.12**	**−18.93**	**−2.72**	**<0.01**
**DACOBS ATB**		**0.40**		**0.09**	**0.23**	**0.56**	**<0.001**
**Sex**		**−2.04**		**0.68**	**−3.38**	**−0.70**	**<0.01**
Age		**−**0.00		0.15	**−**0.30	0.30	0.99
	Dependent Variable Model – outcome: CAPE total						
**Constant**		**62.83**		**5.88**	**51.24**	**74.41**	**<0.001**
**PSS-10**		**0.89**		**0.09**	**0.71**	**1.06**	**<0.001**
**DACOBS ATB**		**0.53**		**0.13**	**0.28**	**0.78**	**<0.001**
CISS distraction		0.17		0.10	**−**0.03	0.36	0.09
**Interaction**		**0.03**		**0.01**	**0.00**	**0.05**	**<0.05**
**Sex**		**4.06**		**0.98**	**2.13**	**6.00**	**<0.001**
Age		**−**0.02		0.23	**−**0.44	0.40	0.96

n = 275; R^2^ for Mediator Model = 0.10, p < 0.001; R^2^ for Dependent Variable Model = 0.40, p < 0.001; CAPE total, total score of the Community Assessment of Psychic Experiences; PSS-10, total score of Perceived Stress Scale; DACOBS ATB, Attention to Threat Bias subscale score of the DACOBS; CAPE, total score of the Community Assessment of Psychic Experiences; CISS distraction, score of subscale of Coping Inventory for Stressful Situations measuring distraction seeking coping style; women were coded as **−**1, men were coded as 1.

The statistically significant results were written in bold.

Importantly, the conditional indirect effects of attention to threat bias on CAPE total *via* stress differed depending on the level of this particular coping style (Index of moderated mediation=0.01, SE=0.01, CI_low_=0.001, CI_high_=0.03). The indirect effect was weaker at the lower levels (Mean – 1 SD) of distraction seeking, and stronger at the higher levels of distraction seeking (Mean, Mean + 1 SD) (**Table 5**). Further analyses showed that the aforementioned effect was limited to positive symptoms of PLEs (CAPE positive) and depression symptoms (CAPE depression); it was not present in the case of negative symptoms of PLEs (CAPE negative) (see **Tables 5** and **6** for details).

**Table 5:** Conditional indirect effects of attention to threat bias on CAPE at values of the CISS distraction.

Values of the CISS distraction	Effect	*SE*	Bootstrap (*n*=5,000)	
			95% CI_low_	95% CI_high_
Dependent variable: CAPE total				
Mean **−** 1SD	0.29	0.07	0.16	0.44
Mean	0.35	0.08	0.21	0.52
Mean+1SD	0.42	0.10	0.25	0.63
Dependent variable: CAPE positive				
Mean **−** 1SD	0.05	0.02	0.01	0.10
Mean	0.08	0.02	0.04	0.13
Mean+1SD	0.11	0.03	0.05	0.18
Dependent variable: CAPE depression				
Mean **−** 1SD	0.12	0.03	0.07	0.19
Mean	0.14	0.03	0.08	0.21
Mean+1SD	0.15	0.04	0.08	0.25

n = 275; CAPE total, total score of the Community Assessment of Psychic Experiences; CAPE negative, negative symptoms subscale of the Community Assessment of Psychic Experiences; CAPE positive, positive symptoms subscale of the Community Assessment of Psychic Experiences; CAPE depression, depression subscale of the Community Assessment of Psychic Experiences; CISS distraction, score of subscale of Coping Inventory for Stressful Situations measuring distraction seeking coping style.

**Table 6:** Conditional indirect effects of cognitive biases on different dimensions of CAPE.

	Index of moderated mediation	*SE*	Bootstrap (*n*=5,000)	
			95% CI_low_	95% CI_high_
Moderator: task-oriented coping				
DACOBS ATB→CAPE total	**−**0.005	0.004	**−**0.013	0.003
DACOBS ATB→CAPE positive	**−**0.001	0.002	**−**0.003	0.003
DACOBS ATB→CAPE negative	**−**0.003	0.002	**−**0.007	0.001
**DACOBS ATB→CAPE depression**	**−0.002**	**0.001**	**−0.004**	**−0.000**
DACOBS ETB→CAPE total	**−**0.008	0.006	**−**0.020	0.003
DACOBS ETB→CAPE positive	**−**0.000	0.002	**−**0.005	0.004
DACOBS ETB→CAPE negative	**−**0.005	0.003	**−**0.012	0.001
**DACOBS ETB→CAPE depression**	**−0.003**	**0.002**	**−0.006**	**−0.000**
Moderator: emotion-oriented coping				
DACOBS ATB→CAPE total	0.002	0.003	**−**0.004	0.008
DACOBS ATB→CAPE positive	0.000	0.002	**−**0.003	0.003
DACOBS ATB→CAPE negative	**−**0.000	0.001	**−**0.003	0.002
**DACOBS ATB→CAPE depression**	**0.002**	**0.001**	**0.000**	**0.004**
DACOBS ETB→CAPE total	0.001	0.005	**−**0.007	0.011
DACOBS ETB→CAPE positive	**−**0.000	0.002	**−**0.005	0.004
DACOBS ETB→CAPE negative	**−**0.001	0.002	**−**0.005	0.004
DACOBS ETB→CAPE depression	0.002	0.001	**−**0.000	0.005
Moderator: distraction seeking				
**DACOBS ATB→CAPE total**	**0.010**	**0.005**	**0.001**	**0.023**
**DACOBS ATB→CAPE positive**	**0.005**	**0.003**	**0.001**	**0.011**
DACOBS ATB→CAPE negative	0.002	0.003	**−**0.003	0.008
**DACOBS ATB→CAPE depression**	**0.003**	**0.002**	**0.001**	**0.007**
DACOBS ETB→CAPE total	0.014	0.008	**−**0.001	0.031
DACOBS ETB→CAPE positive	0.006	0.004	**−**0.001	0.015
DACOBS ETB→CAPE negative	0.003	0.005	**−**0.006	0.013
DACOBS ETB→CAPE depression	0.004	0.003	**−**0.000	0.010
Moderator: social contacts				
DACOBS ATB→CAPE total	0.002	0.008	**−**0.014	0.017
DACOBS ATB→CAPE positive	0.005	0.004	**−**0.002	0.015
DACOBS ATB→CAPE negative	**−**0.002	0.004	**−**0.010	0.005
DACOBS ATB→CAPE depression	**−**0.002	0.002	**−**0.007	0.002
DACOBS ETB→CAPE total	0.000	0.012	**−**0.024	0.023
DACOBS ETB→CAPE positive	0.007	0.006	**−**0.006	0.020
DACOBS ETB→CAPE negative	**−**0.002	0.006	**−**0.014	0.010
DACOBS ETB→CAPE depression	**−**0.005	0.004	**−**0.012	0.020

n = 275; since the presented effects are small, all values are shown to three decimal places; DACOBS ATB, Attention to Threat Bias subscale score of the DACOBS; DACOBS ETB, External Attribution Bias subscale score of the DACOBS; CAPE total, total score of the Community Assessment of Psychic Experiences; CAPE negative, negative symptoms subscale of the Community Assessment of Psychic Experiences; CAPE positive, positive symptoms subscale of the Community Assessment of Psychic Experiences; CAPE depression, depression subscale of the Community Assessment of Psychic Experiences.

The statistically significant results were written in bold.

No other significant moderated mediation effects were detected when the total score of the CAPE scale was treated as a dependent variable. Nevertheless, when additional analyses were conducted separately for the CAPE subscales, a couple more interesting effects emerged concerning the depression symptoms subscale (**Table 6**). It turned out that conditional indirect effect of attention to threat bias *via* perceived stress differed depending on the level of emotion-oriented coping (Index of moderated mediation=0.002, SE=0.001, CI_low_=0.0003, CI_high_=0.004) and task-oriented coping (Index of moderated mediation=−0.002, SE=0.001, CI_low_=−0.004, CI_high_=−0.0003) in such a way that the effect was stronger at the higher level of emotional coping and at the lower level of task-oriented coping (**Table 7**).

**Table 7:** Conditional indirect effects of cognitive biases on CAPE depression at values of the moderators.

	Values of the moderator	Effect	*SE*	Bootstrap (*n*=5,000)	
				95% CI_low_	95% CI_high_
Moderator: emotion-oriented coping					
Predictor: DACOBS ATB	Mean − 1SD	0.09	0.02	0.05	0.13
	Mean	0.11	0.02	0.06	0.16
	Mean+1SD	0.13	0.03	0.07	0.19
Moderator: task-oriented coping					
	Mean − 1SD	0.15	0.03	0.10	0.22
	Mean	0.13	0.03	0.08	0.19
	Mean+1SD	0.12	0.03	0.07	0.17
Predictor: DACOBS ETB	Mean − 1SD	0.24	0.04	0.18	0.32
	Mean	0.21	0.03	0.16	0.27
	Mean+1SD	0.18	0.03	0.13	0.24

n = 275; CAPE depression, depression subscale of the Community Assessment of Psychic Experiences; DACOBS ATB, Attention to Threat Bias subscale score of the DACOBS; DACOBS ETB, External Attribution Bias subscale score of the DACOBS.

#### The Relationship Between External Attribution Bias, Stress, Coping, and PLEs

When ETB was treated as predictor in the moderated mediation models, none of the examined interaction effects of stress and coping on total score of the CAPE reached the level of statistical significance: task-oriented coping × stress: B=−0.09, SE=0.06, CI_low_=−0.20, CI_high_=0.03, p=0.15; emotion-oriented coping × stress: B=−0.01, SE=0.04, CI_low_=−0.09, CI_high_=0.07, p=0.84; avoidant coping × stress: B=0.06, SE=0.06, CI_low_=−0.05, CI_high_=0.17, p=0.29; distraction seeking × stress: B=0.12, SE=0.09, CI_low_=−0.05, CI_high_=0.31; social diversion coping × stress: B=0.08, SE=0.13, CI_low_=−0.18, CI_high_=0.34, p=0.53. Interestingly, when the CAPE subscales were taken into consideration, the conditional indirect effect of external attribution bias on depression turned out to be stronger at the lower level of task-oriented coping (**Tables 6** and **7**).

## Discussion

The current study examined the interrelationship between cognitive biases, perceived stress, and psychotic-like experiences in a sample of healthy young and middle adults (34). It also further explored whether the individual strategies applied to copy with stressful events moderate the link between stress and PLEs. The findings confirmed that a higher level of psychotic-like experiences is associated with oversensitivity to threat (ATB) as well as a tendency to blame others for negative events (ETB). The study also yielded a positive, correlational relationship between stress and biased cognitive processes, and demonstrated that individuals with higher psychological stress are more likely to endorse psychotic-like experiences. These results contribute to growing literature highlighting the importance of cognitive biases and stressful life events for psychotic symptoms within the psychosis continuum (4–6, 10, 11, 13, 15–18).

In the study, we also examined whether the relationship linking oversensitivity to threat and the tendency to blame others for failures with subclinical psychotic symptoms may be explained by the level of stress experienced by individuals. Indeed, the results showed that the relationship linking ATB and ETB with different dimensions of psychotic-like experiences is partially mediated by perceived stress. It suggests that searching environment for threat, social threat in particular, may exaggerate psychotic symptoms through increasing the stress level. This finding provides additional evidence in a non-clinical sample supporting the recent models of psychosis emphasizing the interplay between cognitive and emotional distortions in symptoms development (24, 25). However, it should be noted that the mediation effect yielded in the study was only partial, suggesting that there are some other factors through which cognitive biases may affect psychotic symptoms. For example, the tendency to attribute negative events to external, personal causes may trigger anger or result in social withdrawal and loss of social support, which may likewise lead to symptoms amplification.

In the study, we also considered the role of coping strategies on the relationship between stress and psychotic-like experiences. The link between coping and psychotic experiences has already been examined in a few studies concerning coping methods preferred by individuals with PLEs, UHR subjects, and patients with schizophrenia (16–18, 22). In general, it was demonstrated that individuals with psychotic symptoms reveal the tendency to apply non-adaptive, emotion-focused coping style. The current study also yielded the results suggesting that PLEs are positively related to emotion-oriented and distraction type of avoidance-oriented coping (with the exception of the negative symptoms of PLEs), however, not to social diversion coping. We also observed the weak, negative relationship between the negative symptoms of PLEs as well as depression and task-oriented coping. This coping fashion roughly mirrors the pattern previously found among individuals with clinical psychosis and UHR subjects (17, 22) providing future evidence that the tendency to overuse maladaptive coping may be observed in both subclinical and clinical areas of the psychosis continuum. Similarly, as previously stated in case of a clinical group (22), individuals reporting PLEs may perceive stressful events as uncontrollable and social support as unavailable due to their suspicious and persecutory beliefs. Therefore, they may consider emotion-oriented coping as more adequate than task-oriented one. Also, negative symptoms, such as amotivation, anhedonia and withdrawal, or depression-related hopelessness can be a source of participants' tendency to refrain from acting actively when facing stressful events. Individuals experiencing such symptoms may consider task-oriented coping as too demanding, which makes them willing to apply strategies focused on emotional responses, self-preoccupation, or fantasizing. Also avoidance-oriented coping, such as distraction seeking and social diversion may be difficult to apply for individuals with negative symptoms since these require engaging in new activities and maintaining social contacts.

Although on the basis of our study we cannot draw conclusion about the direction of the relationship linking emotion-oriented coping and PLEs, it is probable that the tendency to apply emotion-focused methods may amplify psychotic-like experiences, which in turn increase the use of emotion-oriented coping. This conclusion is consistent with the findings obtained by Lin et al. (18) in a longitudinal study on a non-clinical sample of adolescents, demonstrating that greater use of emotion-oriented coping is associated with an increase of subclinical psychotic symptoms over time, and that higher level of PLEs at baseline predicted greater use of emotion-focused coping three years later.

The current study also demonstrated that the association between perceived stress and psychotic-like experiences is modified by the coping styles. This finding is in line with previous studies considering the role of coping method in symptoms development and outcome (18, 23). However, contrary to prior research, the present study investigated the role of coping in a broad context of interrelationships between stress and cognitive biases. Particularly, we tested whether individual coping methods may moderate the associations linking cognitive biases (ATB and ETB), stress, and PLEs. We found that the stress-mediated indirect effect of ATB on positive and depressive symptoms of PLEs is stronger at the higher level of distraction seeking type of avoidance coping, and weakens when the tendency to engage in distraction-based activities is reduced. It is likely that in individuals characterized by enhanced threat sensitivity, the use of these strategies may increase psychotic symptoms, since for them distraction-based activities are the source of additional stress. It is also possible, that individuals seeking distraction to cope with stress originating from their increased sensitivity are especially susceptible to psychotic symptoms, since this coping method may prevent them from deeper processing of information they receive from the environment. As a result, they have fewer opportunities to correct their reasoning and are more prone to the harmful effect of cognitive biases. Furthermore, avoidant style of coping keeps them from dealing directly with demands created by the stressful events and as a result may lead to the escalation of a problem, which would start to impact them even more strongly—leading to depressive symptoms.

It should be noted, that the previous study of Chisholm et al. (35) did not find avoidant coping to be maladaptive in a group of non-clinical adolescents. However, Chisholm et al. (35) considered jointly the two types of avoidant styles, i.e. distraction seeking and social diversion, therefore, they were unable to capture the role of each of these styles in the relationship between stress and PLEs. Our findings indicate, that only the level of distraction seeking modifies the stress-PLEs relationship. What is more, in the Chisholm et al. (35) study the role of attention to threat bias was not considered. It is plausible, that distraction seeking increases the PLEs only among distressed individuals with heightened threat sensitivity. For these participants, engaging in distraction-biased activities may provide additional stress due to their tendency to examine the phenomena they encounter in terms of threat, whereas in adolescents without ATB undertaking additional activities may be even adaptive since in adolescents avoidance-oriented coping was found to be positively associated with social relationships (35).

Our study also yielded the moderation effect concerning emotion-oriented coping: the indirect effect of ATB *via* perceived stress on depressive symptoms was stronger at the higher level of emotion-oriented coping. The fact that this effect was not present in regard to positive and negative symptoms of PLEs is somewhat surprising in light of previous studies showing that patients with schizophrenia, UHR subjects and healthy people reporting PLEs are particularly likely to employ emotion-oriented strategies to cope with stressful events (16, 22). It suggests, that although focusing on one's own emotional responses is common among individuals with psychotic experiences, applying this coping method not necessarily shapes the relationship between stress and positive or negative psychotic symptoms. It seems plausible that emotion-oriented coping may be effective as far as individuals with PLEs perceive stress as uncontrollable, i.e. it may decrease the stress level, however, it does not necessarily reduce psychotic experiences. This finding seems to be inconsistent with the previous observation by Lin et al. (18) that emotion-oriented coping predicted PLEs over time. However, in our study we investigated the link between emotion-focused coping and PLEs at a single time point, therefore, our findings do not exclude the possibility that this type of coping is related to some changes in psychotic symptoms that occur over time.

It is noteworthy, that in the current study also task-oriented coping emerged as a moderator shaping the indirect effects of attention to threat bias and external attribution bias on depressive symptoms in such a way, that the effects of perceived stress on depression were weaker at the higher level of task-oriented coping. It is possible that active problem solving protects individuals from depression-related negative consequences of stress such as feeling of helplessness, negative self-esteem, or hopelessness by improving their well-being, perceived efficacy, and sense of control (36). On the other hand, surprisingly, a similar pattern was not found in the case of positive and negative symptoms of PLEs: this finding leaves open the question about the role played by task-oriented coping in reducing psychotic symptoms.

Researching possible factors and mechanisms underlying psychotic-like experiences in non-clinical groups is important for a few reasons. First of all, it has been demonstrated that providing help at a very early stage of disorder emergence and development may prevent, delay, reduce, or help to control later psychotic symptoms (37). Effective prevention needs to take into account not only early symptoms but also and foremost phenomena producing such symptoms and psychotic vulnerability (38). Moreover, studying very early phases of psychosis gives a clearer picture of factors and processes involved, before the development of illness, its consequences, and effects of treatment clouds this picture (38).

The results of our study provide implications for early interventions focused on decreasing subclinical psychotic symptoms through reducing perceived stress or modifying methods used to cope with stressful events. Given that PLEs have been linked to increased risk of psychosis development (1), such interventions should be effective when applied among healthy, at risk individuals in order to preclude or delay the psychosis onset. For example, programs focused on the modification of attention to threat bias as well as on teaching stress alleviation techniques may decrease PLEs by reducing stress. Also, interventions aimed at replacing distraction seeking coping and emotion-focused coping with more adaptive methods may be of assistance to people with heightened threat sensitivity.

The present findings should be interpreted in light of the study limitations. Firstly, variables assessment was based on self-reports and retrospection. It is particularly important in case of psychotic-like experiences which were suggested to be overestimated in self-reports (39). However, evidence also exists, that there is a good correlation between scores obtained on the CAPE questionnaire and interview-based assessment of PLEs (40). Nevertheless, due to the lack of more objective methods for measuring variables, the results obtained in the study should be treated as preliminary. Secondly, the study design was cross-sectional and therefore we cannot establish the causality or directionality of the observed relationships. Moreover, there was no clinical group to assess whether a similar pattern of findings would be found among patients suffering from psychosis or UHR individuals. Furthermore, it may be argued, that excluding participants with family history of psychiatric disorders from the study, although common practice in research concerning psychotic-like experiences, might have unnecessarily limited the variance of measured variables. Future studies with stronger methodologies, such as Experience Samples Methods, and with more heterogeneous as well as clinical groups are needed to confirm the obtained results. Also, the imbalance of the study sample in terms of gender and education level limits the generalizability to the general population. Finally, it is worth mentioning that apart from modifying the relationship between perceived stress and PLEs, it is also possible that coping styles change the way in which cognitive biases affect the subjective appraisal of stress. Although the additional analyses conducted by the authors did not yield significant interaction effects of cognitive biases and coping methods on perceived stress this model should be explored in further longitudinal studies.

In summary, despite its limitations, the study provided new data concerning the interplay between cognitive biases (widely recognized as contributing to psychotic symptoms), subjective experiences of stress, coping styles, and psychotic-like experiences. The results imply that addressing simple relations between cognitive biases and perceived stress may be insufficient to understand psychotic symptoms. The findings suggest that stress associated with the heightened threat sensitivity may aggravate the psychotic symptoms especially among individuals employing distraction seeking and emotion-oriented coping methods. Therefore, our study provided theoretical basis for early intervention strategies.

## Data Availability Statement

The datasets generated for this study are available on request to the corresponding author.

## Ethics Statement

The studies involving human participants were reviewed and approved by Komisja ds Etyki Badań Naukowych przy Instytucie Psychologii Uniwersytetu Jagiellońskiego. The patients/participants provided their written informed consent to participate in this study.

## Author Contributions

KP and JK designed the study. AD gathered data. JK run and described the statistical analyses. KP and JK interpreted the data and wrote the manuscript. All authors approved the final version of the manuscript. All the authors edited the manuscript.

## Conflict of Interest

The authors declare that the research was conducted in the absence of any commercial or financial relationships that could be construed as a potential conflict of interest.
